# Supplementary material for: Adaptation Strategies of Halophytic Barley Hordeum marinum ssp. marinum to High Salinity and Osmotic Stress
Source: Int J Mol Sci. 2020 Nov 27;21(23):9019. doi: 10.3390/ijms21239019 (PMC7730945; doi:10.3390/ijms21239019)
Supplement: Supplementary file 1 [file ijms-21-09019-s001.zip › Supplementary Figure S1.pptx]

## Slide 1
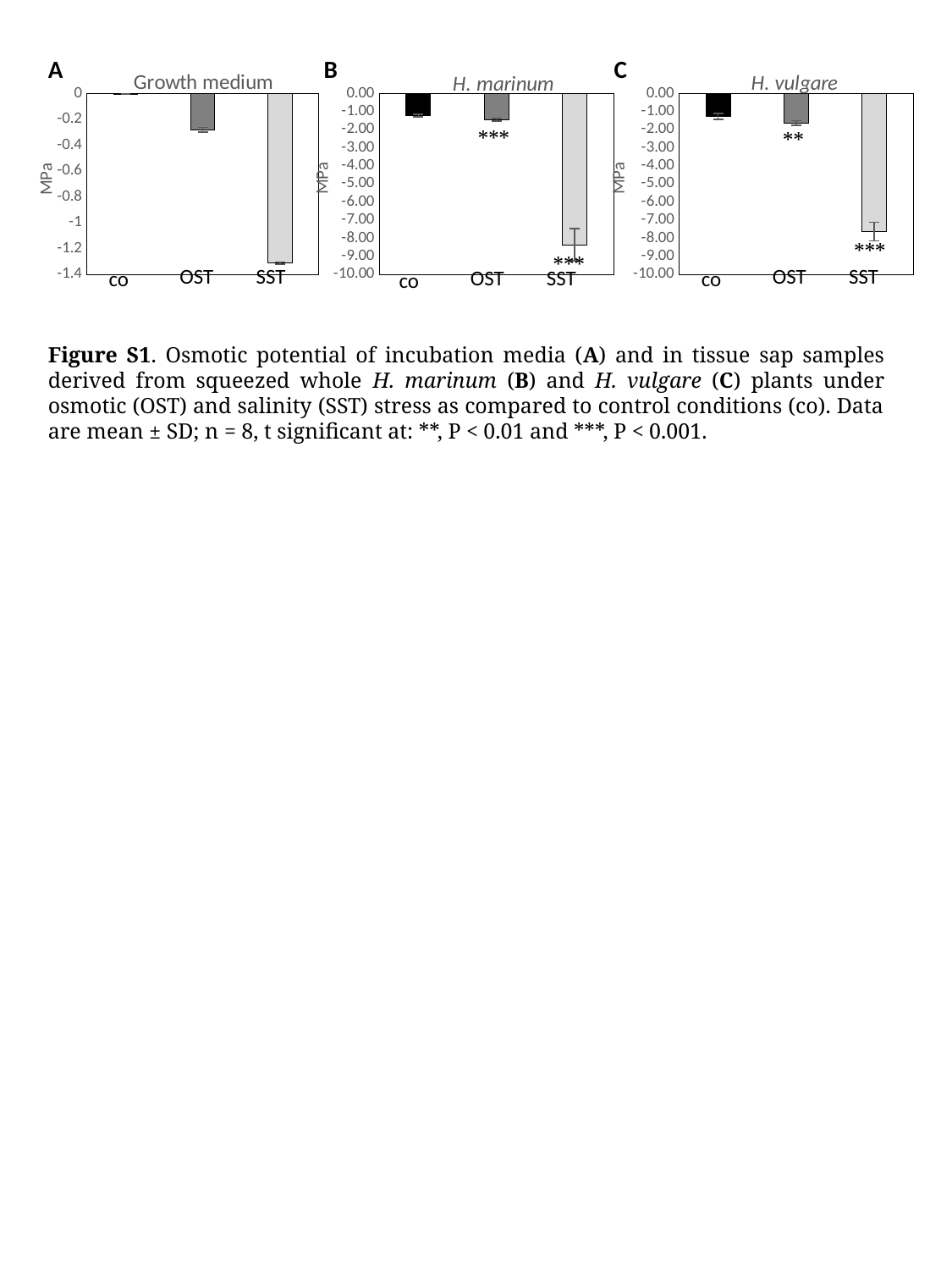

### Chart: Growth medium
| Category | |
|---|---|
| Control | -0.0021 |
| 15% PEG6000 | -0.28093 |
| 300 mM NaCl | -1.31322 |
### Chart: H. marinum
| Category | |
|---|---|
| Control | -1.22 |
| 15% PEG6000 | -1.4577 |
| 300 mM NaCl | -8.3633 |
### Chart: H. vulgare
| Category | |
|---|---|
| Control | -1.27 |
| 15% PEG6000 | -1.63 |
| 300 mM NaCl | -7.6282 |C
A
B
***
**
***
***
co
co
co
OST
SST
OST
SST
OST
SST
Figure S1. Osmotic potential of incubation media (A) and in tissue sap samples derived from squeezed whole H. marinum (B) and H. vulgare (C) plants under osmotic (OST) and salinity (SST) stress as compared to control conditions (co). Data are mean ± SD; n = 8, t significant at: **, P < 0.01 and ***, P < 0.001.
