# Supplementary material for: Adaptation Strategies of Halophytic Barley Hordeum marinum ssp. marinum to High Salinity and Osmotic Stress
Source: Int J Mol Sci. 2020 Nov 27;21(23):9019. doi: 10.3390/ijms21239019 (PMC7730945; doi:10.3390/ijms21239019)
Supplement: Supplementary file 1 [file ijms-21-09019-s001.zip › Supplementary Figure S2.pptx]

## Slide 1
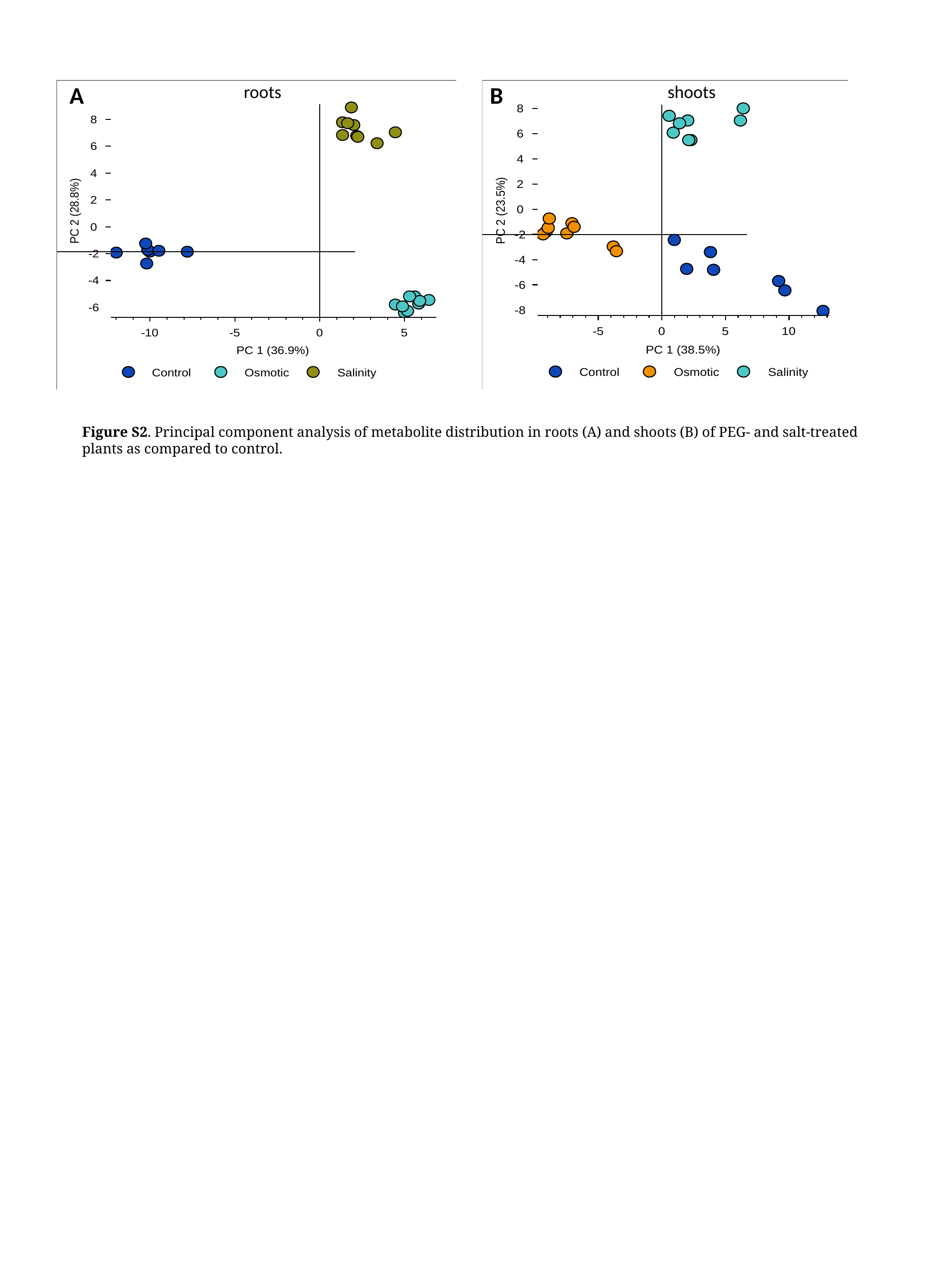

A
roots
B
shoots
Figure S2. Principal component analysis of metabolite distribution in roots (A) and shoots (B) of PEG- and salt-treated plants as compared to control.
