# Supplementary material for: Adaptation Strategies of Halophytic Barley Hordeum marinum ssp. marinum to High Salinity and Osmotic Stress
Source: Int J Mol Sci. 2020 Nov 27;21(23):9019. doi: 10.3390/ijms21239019 (PMC7730945; doi:10.3390/ijms21239019)
Supplement: Supplementary file 1 [file ijms-21-09019-s001.zip › Supplementary Table S3.docx]

**Table S2.** Chromatographic and Mass spectrometry conditions for the untargeted metabolite analysis

| **Ion chromatography** | **Mass Spectrometry** |
| --- | --- |
| Analytical columns:  Dionex™ IonPac™ AS11-HC-4µm (2x250 & 2x50mm)  Eluents:  A: H2O  B: 100mM KOH (generated by Dionex EGC 500 KOH cartridge)  Gradient:  Time %B  0-12 10-25  12-15 25-100  15-28 100  28-28.5 100-10  28.5-32 10  Flow parameters:  Flow 0.35 mL/min  Temp 35 °C  Inj. Vol 10 µL | Tune parameters:  Polarity: negative  Spray voltage: 3.5 kV  Sheath gas flow: 36  Aux. gas flow: 5  Capillary temp.: 320 °C  Aux. gas temp.: 325 °C  S-lens: 50  Acquisition mode: Full MS  Time: 0-30 min  Resolution: 140 K  m/z Range: 67 - 1000  Inject time: 200 ms  Auto gain control: 3e6  Acquisition mode: Full MS / ddMS2  Time: 0-30 min  FullMS:  Resolution: 70 K  m/z Range: 67 - 1000  Inject time: 100 ms  Auto gain control: 1e6  dd-MS2:  Resolving power: 17.5 K  Inject time: 50 ms  Auto gain control: 1e5  Loop count: 5  Collision energy: 15; 25; 35 |
